# Hot and Cold Tumors: Is Endoglin (CD105) a Potential Target for Vessel Normalization?

**DOI:** 10.3390/cancers13071552

**Published:** 2021-03-28

**Authors:** Claudia Ollauri-Ibáñez, Blanca Ayuso-Íñigo, Miguel Pericacho

**Affiliations:** Renal and Cardiovascular Research Unit, Group of Physiopathology of the Vascular Endothelium (ENDOVAS), Biomedical Research Institute of Salamanca (IBSAL), Department of Physiology and Pharmacology, University of Salamanca, 37007 Salamanca, Spain; collauri@usal.es (C.O.-I.); blancayuso97@usal.es (B.A.-Í.)

**Keywords:** endoglin, CD105, angiogenesis, tumor microenvironment, inflammation, vessel normalization

## Abstract

**Simple Summary:**

The prognosis and response to immunotherapy depends largely on the composition of the tumor microenvironment (TME). So-called cold tumors are rich in cells and molecules that inhibit the antitumor response and, therefore, are associated with a worse prognosis. In contrast, hot tumors are rich in antitumor cells and respond well to immunotherapy. The creation of one type of TME or another is highly dependent on angiogenesis, inflammation, and cancer-associated fibroblast (CAF) accumulation. Endoglin (CD105) is a protein involved in these three processes, making it a possible target for the conversion of cold tumors into hot tumors. In this review we summarize the role of endoglin in these processes and present the anti-endoglin therapies already under study that could be applied for vascular normalization.

**Abstract:**

Tumors are complex masses formed by malignant but also by normal cells. The interaction between these cells via cytokines, chemokines, growth factors, and enzymes that remodel the extracellular matrix (ECM) constitutes the tumor microenvironment (TME). This TME can be determinant in the prognosis and the response to some treatments such as immunotherapy. Depending on their TME, two types of tumors can be defined: hot tumors, characterized by an immunosupportive TME and a good response to immunotherapy; and cold tumors, which respond poorly to this therapy and are characterized by an immunosuppressive TME. A therapeutic strategy that has been shown to be useful for the conversion of cold tumors into hot tumors is vascular normalization. In this review we propose that endoglin (CD105) may be a useful target of this strategy since it is involved in the three main processes involved in the generation of the TME: angiogenesis, inflammation, and cancer-associated fibroblast (CAF) accumulation. Moreover, the analysis of endoglin expression in tumors, which is already used in the clinic to study the microvascular density and that is associated with worse prognosis, could be used to predict a patient’s response to immunotherapy.

## 1. Introduction

Cancer is a major public health problem and is the second leading cause of death in developed countries. The mortality rate increased until 1991 and then began to decrease. This decrease is mainly due to the reduction of mortality from lung, colorectal, breast, and prostate cancer. However, this reduction has slowed in breast and colorectal cancer and stagnated in prostate cancer. Thus, Siegel and colleagues estimated that in 2020 there would be 1,806,590 new cases and 606,520 deaths in the United States alone [1].

In 2000, Hanahan and Weinberg proposed six hallmarks or capabilities that allowed tumor cells to survive, proliferate, and spread: (1) Sustaining proliferative signaling; (2) evading growth suppressors; (3) resisting cell death; (4) activating invasion and metastasis; (5) enabling replicative immortality; and (6) inducing angiogenesis [2]. In 2011 they added two new enabling characteristics and two hallmarks. The enabling characteristics included chromosomal instability and the inflammatory status of the premalignant lesion, which may determine whether or not the tumor develops. The new hallmarks were the reprogramming of cell energy metabolism towards mechanisms that consume less oxygen and avoid immune destruction. The immune system eliminates most of the potentially cancerous cells, so those that manage to generate a solid tumor are able to evade this response. In addition, immune cells and inflammation can contribute to the development of several hallmarks by, for example, releasing bioactive molecules that promote proliferation and survival and reduce cell death, proangiogenic factors, enzymes that modify extracellular matrix (ECM) and facilitate angiogenesis, invasion and metastasis, or signals that promote epithelium-mesenchyme transition (EMT). They can also produce reactive oxygen species (ROS) that promote metastasis in nearby tumor cells [3,4].

For all these reasons, tumors cannot be considered simple masses of abnormal cells in proliferation, but rather complex tissues composed by different types of cancerous and normal cells which interact with each other [4,5]. In this review we will define the concept of tumor microenvironment (TME) and explain how it can affect the patient’s prognosis according to the cell types and factors that determine it. We will explain the concept of vascular normalization and its effects on TME. Finally, we, will present endoglin as a key protein in the formation of a more immunosuppressive tumor microenvironment and, therefore, as a possible biomarker of this type of TME and a possible therapeutic target to, through vascular normalization, generate a more immunosupportive TME.

## 2. Tumor Microenvironment (TME)

The TME is created by interactions between tumor cells and normal or non-transformed cells that form the tumor mass [5]. Intercellular communication takes place through a complex network of cytokines, chemokines, growth factors, and enzymes that remodel ECM [5], constituting a dynamic, acidified, and heterogeneous space that can determine a better or worse prognosis or even be responsible for the success or failure of treatments [6,7], especially in the case of immunotherapy.

### 2.1. Non-Cancerous Cellular Component of the TME

**Figure 1:**
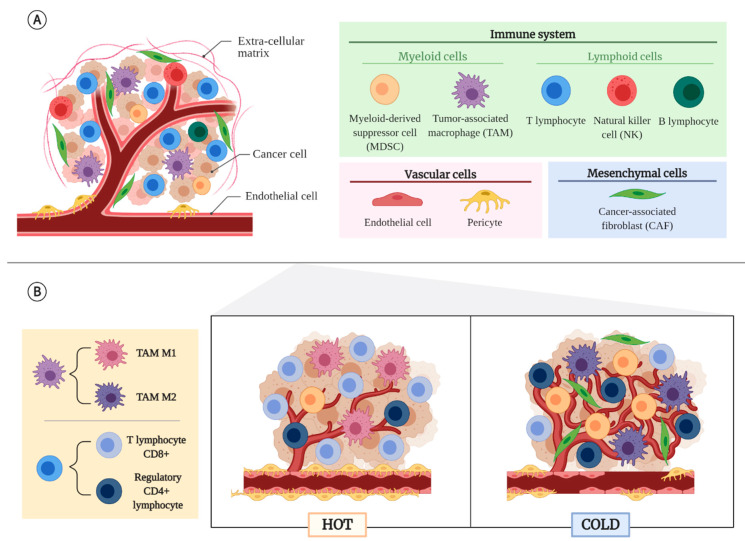
**Tumor microenvironment (TME)**. (**A**) **Origins of non-cancerous cells of the TME**. Non-malignant cells that form TME can be classified according to their origin as immune cells, mesenchymal cells, and vascular cells. Immune cells can also be divided in lymphoid, including T and B lymphocytes or natural killers (NKs), or myeloid cells, including tumor-associated macrophages (TAMs) or myeloid-derived suppressor cells (MDSCs). Cancer-associated fibroblasts (CAFs) are the main cell of mesenchymal origin in tumors. Endothelial cells (ECs) and pericytes form blood vessels of the tumor. (**B**) **Tumor types according to their TME**. Hot tumors are enriched in CD8^+^ lymphocytes and M1 TAMs and present low infiltration of MDSCs or CAFs resulting in a better response to immunotherapy. On the other hand, cold tumor TME are characterized by the presence of more Treg than CD8^+^ lymphocytes and high infiltration of M2 TAMs, MDSCs, and CAFs. Moreover, cold tumors present worst vasculature than hot tumors and they generally respond very poorly to immunotherapy. Created with BioRender.com.

The non-malignant cells that form the TME come from different lineages or origins: immune cells, cells of mesenchymal origin, and vascular cells [5,6,8,9] (Figure 1A).

Cells from the immune system can be classified according to their origin between lymphoid or myeloid lineage. Among the lymphoid cells, we find cytotoxic CD8^+^ lymphocytes, which have an antitumor function, so a higher infiltration is related to a good prognosis [5,6]. On the contrary, regulatory CD4^+^ lymphocytes (Tregs) are the immunosuppressive cells par excellence. In physiological conditions these cells have a key role in preventing autoimmunity, but in cancer they promote tumor growth by releasing protumor and anti-inflammatory cytokines, such as interleukin 10 (IL-10) or transforming growth factor β (TGF-β), and proangiogenic molecules, such as vascular endothelial growth factor (VEGF) [5,6,10,11]. Moreover, Tregs express the receptor CTLA-4, which acts as an inhibitor of the antitumor response carried out by CD8^+^ lymphocytes [5,12]. Also, in the lymphoid lineage, but forming part of the innate immune response, natural killer cells (NKs) stand out, which also have antitumor function and are related to a good prognosis [5]. B-lymphocytes can be found at the tumor edge, although they are more commonly found in the lymph nodes near the tumor. In ovarian and breast cancer their presence is related to good prognosis [5].

Among cells of myeloid origin, which are mostly recruited by hypoxia [13], the tumor-associated macrophages (TAMs) have special relevance. They can come from tissue-resident macrophages or be derived from monocytes. Regardless of their origin, there are two subpopulations of TAMs: classically activated macrophages (M1), which are immunostimulant; and alternatively activated macrophages (M2), which are immunosuppressive and protumor. In fact, TAMs with M2 phenotype play an important role in tumor cell migration, invasion, and metastasis [10,12,14]. In addition to TAMs, there are myeloid-derived suppressor cells (MDSCs) that are capable of inhibiting the function of effector cells and inducing the enrichment in Treg lymphocytes and the polarization of TAMs towards an M2 phenotype [5,10].

Cancer-associated fibroblasts (CAFs) are the most important and frequent cells with mesenchymal origin in tumors. They can be derived from fibroblast of nearby connective tissues, endothelial cells (ECs), vascular smooth muscle cells, myoepithelial cells, and local or bone marrow mesenchymal stromal cells (MSCs), among others [3,5,15]. The population of CAFs in solid tumors consists of different subtypes of CAFs that may respond differently to stimuli, have very different secretory phenotypes, and exert different roles within the TME. Although no definitive consensus has yet been reached on the nomenclature to be used with these CAF subtypes [16], they are considered to be mainly pro-tumorigenic because of their release of cytokines and chemokines such as IL-6, CXCL9, or TGF-βb that reduce the activity of cytotoxic T lymphocytes [17,18]. However, there are also anti-tumor CAFs that secrete factors that stimulate the immune system [18] or form a physical barrier that restricts tumor cell growth and migration [19]. In some tumors they are located in fibrovascular nuclei that branch out from the tumor, while in others they surround the tumor cells, occupying most of the stroma and making it difficult for the cytotoxic drugs to reach the malignant cells. A higher density of CAFs is also observed on the invasive front of the tumor [5].

Among the vascular cells, we must highlight the ECs and pericytes. The main role of these cells is the formation of blood vessels that provide oxygen and nutrients to the tumor, but they also regulate the flow of inflammatory cells into the tumor stroma [3] and of tumor cells into the bloodstream, facilitating metastasis [3,5]. In that sense, a low mural coverage with pericytes is related to more permeability and metastases and, therefore, worse prognosis [5].

### 2.2. Tumor Types According to Their TME

Tumors can be divided into two large groups according to the characteristics of their TME: hot and cold (Figure 1B). Hot or inflammatory tumors are those that contain a high infiltration of CD8^+^ T lymphocytes and M1 TAMs, and a low infiltration of MDSCs [14,20]. Moreover, high concentrations of chemokines that favor the recruitment of this type of lymphocyte, such as CCL5, CXCL9, and CXCL10, and high expression of interferon γ (IFN-γ), that support their functionality, have been found [20]. This type of TME is called immunosupportive, infiltrated-inflamed, and T-cell infiltrated [12,14,21,22]. Previously these tumors were also called immunogenic, since they express mutated epitopes or neoepitopes as a result of a great chromosomal instability [12,20]. They usually show a good response to immunotherapy [20].

Cold or non-inflammatory tumors have a reduced infiltration of cytotoxic lymphocytes, which are located at the edge of the tumor or sequestered in fibrous areas [12,14,20]. In contrast, they have a large number of Tregs, M2 TAMs, MDSCs, and CAFs [14,20]. This type of TME is called immunosuppressive, infiltrated-excluded, or non-T cell inflamed [12,14,21,22]. Tumors with this type of TME usually have a low mutational load and generally respond very poorly to immunotherapy [6,20].

### 2.3. Factors That Determine an Immunosuppressive TME

There are different factors that make TME immunosuppressive. Firstly, as already mentioned, this type of TME is infiltrated by a large number of cells that inhibit the antitumoral activity of the immune system [12,14,20,21,22]. These cells provide or stimulate the production of factors that increase the proliferation of tumor and stromal cells and inhibit the recruitment and activation of effector cells. They also release enzymes and proteases that modify the structure and function of the ECM. During this modification of ECM, proteases can liberate mitogenic factors and other substances that increase tumor cell migration. In addition, this migration is favored by a less dense ECM. Proteases can also break the cell–cell and cell–ECM bonds that produce the so-called contact inhibition, so tumor cells avoid this suppression. Although the absence of cell–cell contacts would stimulate cell death, the binding of tumor cells to M2 TAMs through integrins prevents this death. Furthermore, it has been shown that substances like RANKL, expressed by TAMs and T cells, suppress the transcription of genes that inhibit metastasis, such as maspin [23]. Another important protein highly present in the TME is autotaxin (ATX), secreted by TAMs, inflamed adipose tissue and cancer cells, among others. This enzyme is an inflammatory mediator that promotes cancer progression and therapy resistance through production of lysophosphatidate (LPA), a phospholipid that increases tumor growth, metastasis, and angiogenesis, and promotes resistance to chemotherapy and radiotherapy. Furthermore, in a pro-inflammatory environment such as the TME, the secretion of inflammatory cytokines and growth factors further induce ATX secretion, creating a cycle that produces more LPA [24]. Besides LPA, another ligand of the lysophospholipid receptor family is sphingosine-1-phosphate (S1P), a lipid mediator which is also released by TAMs and cancer cells. S1P participates in numerous processes such as angiogenesis, proliferation, and migration [25,26]. It is known for its role in the vascular system as a potent angiogenic factor which mediates vascular stability, permeability, and sprouting through its own receptors and also through VEGFR-mediated signaling [27,28]. S1P has also been suggested to have an anti-inflammatory role by inducing a switch from proinflammatory M1 TAMs to anti-inflammatory M2 TAMs [24].

CAFs are also a key part of the definition of the TME. Normal fibroblasts in contact with malignant cells have been shown to prevent tumor cell proliferation. However, they lose this capacity during their transformation into CAFs, making it easier for tumor cells to evade suppression mechanisms [3,15]. They are also known to stimulate EMT, probably by releasing TGF-β, thus increasing invasion and metastasis [29]. Furthermore, like inflammatory cells, CAFs release growth factors that enhance cancer cell proliferation and migration—such as mitogenic epithelial growth factors and TGF-β, respectively—survival factors and proteases that remodel the ECM [15,30]. On the other hand, tumor cells release ROS that make CAFs switch to an aerobic glycolysis that release lactate and pyruvate, which are later used by tumor cells. There are also tumors that use the unesterified fatty acids generated by tumor-associated adipocytes to produce adenosine triphosphate (ATP) by mitochondrial β-oxidation. This type of metabolism protects cells from apoptosis [31].

Another essential factor in the creation of the immunosuppressive microenvironment is angiogenesis. It is known that the formation of new vessels inside tumors facilitates the arrival of oxygen and nutrients and the removal of waste products, allowing tumor growth [32,33,34,35]. However, the imbalance between proangiogenic and antiangiogenic factors in the tumor stroma makes the vessels abnormal, fenestrated, and leaky, facilitating intravasation of tumor cells and metastasis and evasion of anti-tumor surveillance [3,6,32,33,34,35,36]. One of the causes of this imbalance is hypoxia, which stimulates the production of proangiogenic factors [6,33,37]. Moreover, hypoxia promotes EMT and allows the selection of tumor cells capable of surviving in the most unfavorable conditions, through mechanisms like autophagy or the change of metabolism towards types that consume less oxygen, such as aerobic glycolysis [3,6,14,34,38,39]. Finally, it should be noted that abnormal vessels and hypoxia are also related to treatment resistance. The lack of functionality of the vessels prevents the arrival of cytotoxic agents to the tumor cells [6,37,38] and there are treatments such as photodynamic therapy (PDT) that are highly dependent on the presence of oxygen [40], so an increased hypoxia prevents the response.

**Figure 2:**
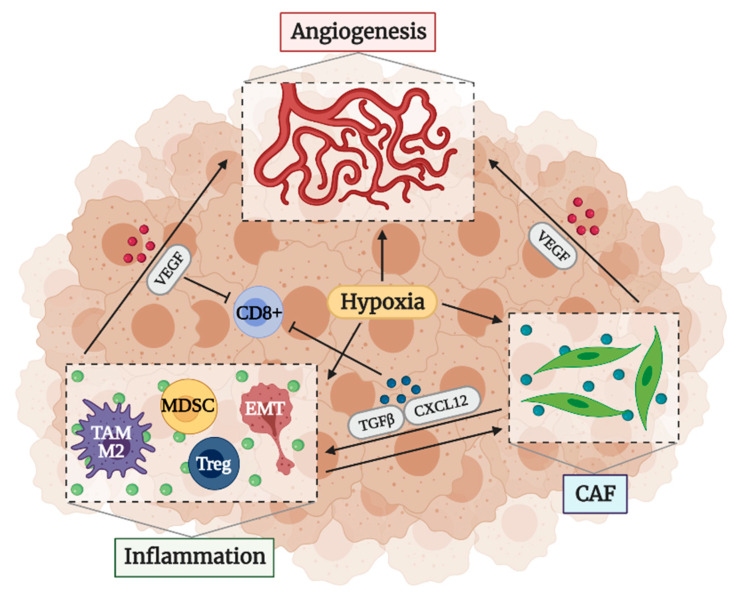
**Factors that determine an immunosuppressive TME: angiogenesis, inflammation, and CAFs**. Inflammatory cells and CAFs stimulate angiogenesis by releasing proangiogenic factors such as vascular endothelial growth factor (VEGF), which also suppress the activity and recruitment of effector cells. Moreover, CAFs also suppress the recruitment of cytotoxic T cells by liberating immunosuppressive ligands and promote the recruitment of other suppressor cells that activate CAFs in turn. On the other hand, hypoxia stimulates angiogenesis, participates with CAFs to maintain the stemness of cancer cells, and induces the recruitment of different types of immunosuppressive cells. Created with BioRender.com.

Although inflammation, CAFs and angiogenesis may seem independent factors, they are interconnected (Figure 2). Both inflammatory cells and CAFs stimulate angiogenesis by releasing different cytokines, ROS, bioactive molecules, and remodeling the ECM, which can liberate proangiogenic factors retained in the matrix [3,6,15]. Moreover, these factors decrease leukocyte–endothelium interactions, reducing the infiltration of effector cells [3,36]. Proangiogenic factors by themselves also suppress the activity and recruitment of effector cells [11,14,36,41]. In addition, hypoxia induces polarization of TAMs to a M2 phenotype and differentiation of MDSCs into ECs and TAMs [6,14]. Hypoxia and CAFs are able to maintain the stemness of cancer cells [6,15]. The relationship between CAFs and inflammation is also evident. CAFs are able to inhibit the activity and recruitment of cytotoxic T cells by releasing immunosuppressive ligands such as TGF-β or CXCL12 [15,42,43], while promoting MDSCs and other suppressor cells recruitment [15,44]. These cells in turn activate CAFs to acquire an inflammatory and protumor phenotype by the liberation of inflammatory modulators like IL-1 or IL-6 [15,45,46]. CAFs can also be activated by different molecules of the ECM [47] and induce the polarization of TAMs towards a M2 phenotype [15,48,49].

## 3. Vascular Normalization as Therapeutic Strategy

Immunotherapy is a growing type of therapy for which Dr. Allison and Dr. Honjo were awarded the Nobel Prize in 2018 [50]. This type of therapy, that mainly includes immune checkpoint inhibitors such as anti-PD-L1/PD-1 or anti-CTLA-4 [51], uses the ability of the immune system’s effector cells to selectively destroy malignant cells, while keeping healthy tissues intact [14,50]. However, it is not enough to increase the number and activity of effector cells since, as mentioned above, an inadequate TME can inhibit their function, so immunotherapy is only successful in a small number of patients [3,11,14,15,36,41]. A strategy that has been shown to be effective in converting an immunosuppressive TME into an immunosupportive TME is target therapy against angiogenesis.

Since Dr. Folkman demonstrated in the 1970s that the presence of blood vessels inside the tumors is essential for their growth [32], a large number of drugs that try to block this process have been developed. These drugs, which mainly target VEGF, FGF, and PDGF pathways [52,53,54], promote the starvation of tumor cells and cell death, increasing tumor regression and patient survival [55]. When the first antiangiogenic inhibitors were developed, it was thought that they might also have some advantages over other drugs that target tumor cells. Firstly, the target cells of antiangiogenic drugs are ECs which, being in contact with blood, are more accessible and, being genetically stable, undergo few changes that could lead to resistances. Furthermore, as most ECs in the body are in a quiescent state and antiangiogenic drugs target active cells, the side effects are less frequent [56].

Unfortunately, despite expectations, clinical benefits are limited, and many patients do not respond or soon develop resistance [11,12,55,56,57]. The main reason of these resistances is that high and prolongated doses of antiangiogenic drugs lead to increased hypoxia and enhanced expression of different proangiogenic molecules and cytokines that results in the recruitment of M2 TAMs, MDSCs, Tregs, and CAFs [11,55,56,57]. On the other hand, tumor vascularization is not only produced by angiogenesis but is also a consequence of other mechanisms such as co-option, vasculogenic mimicry, vessel intussusception, or vasculogenesis from endothelial precursors recruited to the tumor stroma [55,56,57].

**Figure 3:**
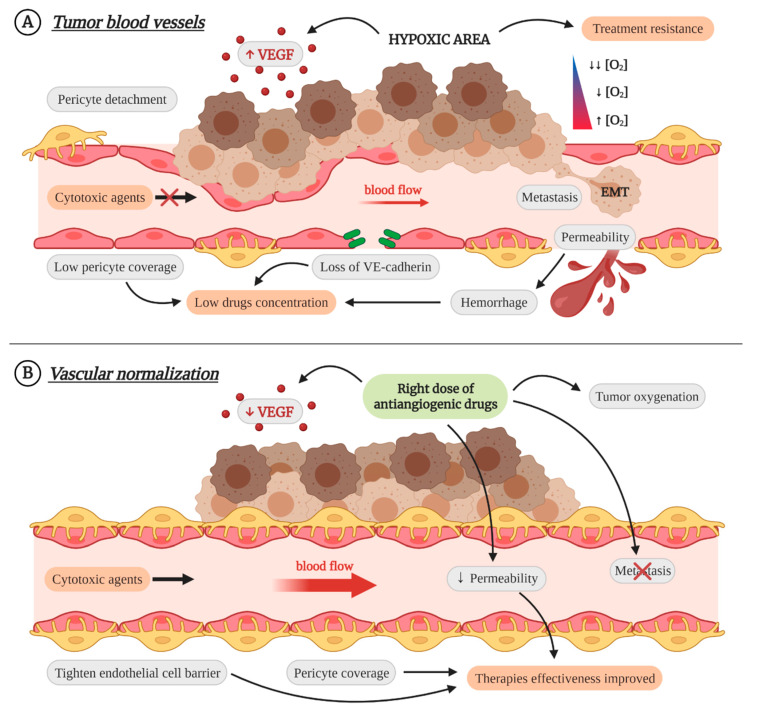
Characteristics and consequences of immature tumor blood vessels, and the effect of vascular normalization as a therapeutic strategy. (**A**) **Tumor blood vessels**. Tumor vasculature is abnormal and with less mural coverage, which increases vascular permeability and leads to the apparition of hemorrhages. The uncontrolled proliferation of tumor cells causes increased hypoxia and allows their intravasation and metastasis. Moreover, the hypoxic microenvironment and permeability prevent the arrival of drugs and cytotoxic agents to large tumor areas and favor the appearance of treatment resistances. (**B**) **Vascular normalization**. The use of the right dose of antiangiogenic drugs avoids blood vessels abnormality, which improves blood flow, reduces hypoxia and permeability, and allows the activity of cytotoxic agents. In consequence, therapy effectiveness is improved and, moreover, it prevents intravasation of tumor cells and metastases. Created with BioRender.com.

Therefore, when it was already thought that the use of antiangiogenic drugs would be short, Dr. Jain proposed that the use of the right dose of antiangiogenics could lead not to the inhibition of blood vessels but to the avoidance of their abnormality. This vascular normalization reduces permeability, preventing the intravasation of tumor cells and metastases [14,55,58]. It also improves blood flow and tumor perfusion, reducing hypoxia, increasing antigen presentation by dendritic cells and M1 TAMs, and promoting the activation of CD8^+^ lymphocytes and the reprogramming of M2 TAMs towards M1 phenotype [11,14,36,55]. That is, vascular normalization can convert a cold tumor into a hot one [11]. Moreover, in combination with other therapies, it improves their effectiveness. The increased perfusion facilitates the arrival of cytotoxic drugs to all points of the tumor and provides the necessary oxygen for some types of radiotherapy [11,40,55]. It has also been shown to increase the effectiveness of immunotherapy [14,55] (Figure 3).

Most of these results have been obtained in pre-clinical and clinical trials using inhibitors of VEGF or its signaling pathway [14,55,58]. However, tumors are very heterogeneous and it may be necessary to target several proangiogenic factors to obtain better results. Thus, it has been shown that anti-angiopoietin, PDGF, and PlGF treatments can also normalize tumor blood vessels [59]. In this review we present the potential to be a target for vascular normalization of another protein traditionally considered as proangiogenic: endoglin.

## 4. Endoglin

Endoglin (CD105) is a type I membrane glycoprotein that acts as coreceptor of TGF-β superfamily [60]. It is mainly expressed in activated ECs [60], but also in CAFs [61,62], MSCs [63], some cancerous cells [64,65,66], and several subpopulations of immune cells [61,67,68,69,70,71,72,73,74,75]. It contains a long extracellular domain, a transmembrane domain, and a short intracellular tail that reflects its function as coreceptor, since it does not initiate the signaling cascade but regulates it. That is, it requires the presence of additional receptors such as TGF-β receptor I kinase (TβRI) and TGF-β receptor II kinase (TβRII) to induce signaling [76]. Depending on the length of this intracellular domain, there are two isoforms of endoglin anchored to the membrane and that are produced by alternative splicing: L-endoglin and S-endoglin. L-endoglin, the majority isoform, has a cytoplasmatic tail of 47 amino acids and is the one most publications refer to. It promotes signaling through ALK1 and Smad1/5/8 [77]. On the other hand, S-endoglin has an intracellular domain of 14 amino acids, of which only half are common with the large isoform. S-endoglin promotes signaling through ALK5 and Smad2/3 [77] and it is associated with aging and cell senescence [78,79,80]. In addition to these two isoforms, there is a soluble form that is produced by cutting the extracellular domain by MMP-14 [81] and MMP-12, in the case of inflammatory macrophages [68]. It is believed that soluble endoglin acts as a trap of TGF-β [82], BMP-9 and BMP-10 [35,83,84]. However, it has been shown that the binding of monodimeric soluble endoglin to BMP-9 does not inhibit its signaling, but potentiates it, although it depends on the presence of membrane endoglin [85]. High levels of soluble endoglin have been found in patients with preeclampsia [86,87] and in some types of cancer, although there is controversy about its function in tumors, since in some cases it seems to have an antitumor role and it is associated with worse prognosis in others [88].

**Figure 4:**
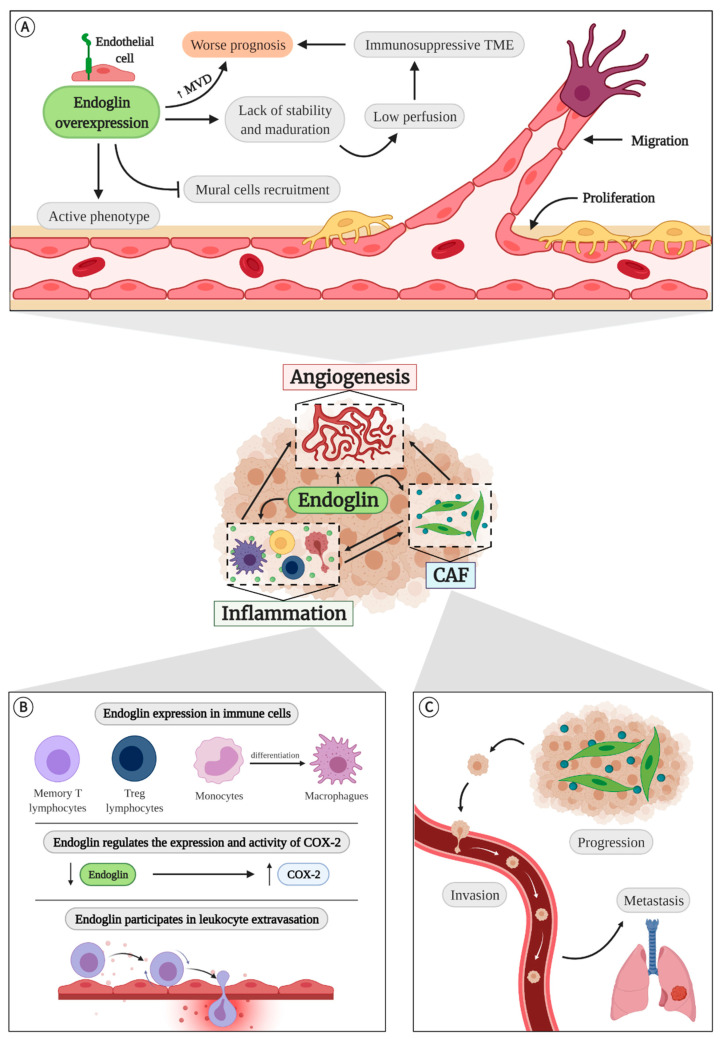
Endoglin plays an important role in the three factors involved in the generation of an immunosuppressive TME: angiogenesis, inflammation, and CAFs. (**A**) **Endoglin in angiogenesis**. Endoglin overexpression maintains an active phenotype in ECs and prevents recruitment of mural cells, which leads to impaired maturation and stabilization of the vessels. This results in low perfusion that could lead to an immunosuppressive microenvironment, which would be responsible for the worse prognosis associated with tumors with high endoglin levels. In fact, endoglin immunohistochemistry is used to measure microvascular density and determinate the tumor prognosis. (**B**) **Endoglin and inflammation**. Endoglin is expressed in different types of immune cells. Moreover, endoglin regulates the expression and activity of COX-2 and participates in leukocyte extravasation. (**C**) **CAFs and endoglin**. Increased endoglin expression in CAFs promotes invasion and metastases. Created with BioRender.com.

Regarding to membrane endoglin, and more specifically to L-endoglin, it has been shown to play a very important role in the three factors mentioned above that are involved in the generation of an immunosuppressive TME: angiogenesis, inflammation, and accumulation of CAFs (Figure 4).

### 4.1. Endoglin in Angiogenesis

One reason that make endoglin a good target to vascular normalization is its involvement in angiogenesis. It has been shown that endoglin expression is increased in the endothelium with active angiogenesis [33]. Specifically, endoglin is overexpressed in the vascular front where sprouting takes place [89,90]. Moreover, several reports show that membrane endoglin also increases in hypoxic conditions such as after myocardial infarction [91], in blood vessels that have suffered vascular damage [92], after induction of retinal angiogenesis [89,93], and during the pathologic angiogenesis of chronic colitis [94] and several solid tumors [95].

In addition, there is other evidence linking endoglin to angiogenesis and vascular remodeling. First, endoglin-deficient mice (*Eng*^−/−^) die during gestation (E10-11.5). These animals have a defective vascular remodeling, which makes vessels fragile and easily broken, resulting in internal bleeding. They also present alterations in the cardiac development, malformations in cardiac valves and in the heart partition [96,97,98]. In the case of zebrafish, homozygous mutants of endoglin are viable and survive to adulthood but have vascular malformations due to the incorrect union of the dorsal artery and cardinal vein [99].

In humans, haploinsufficiency of endoglin is responsible for one of the most common types of hereditary hemorrhagic telangiectasia (HHT) [100,101]. HHT is a rare disease characterized by abundant telangiectasias in the face, hands and oral mucosa, whose rupture results in severe and frequent nosebleeds. HHT patients may also have arteriovenous malformations (AVMs) in the brain, liver, and gastrointestinal tract [101,102,103]. The most used animal model to study HHT caused by endoglin mutations (HHT-1) are *Eng*^+/−^ mice. Contrary to *Eng*^−/−^ mice, heterozygous mice are viable and fertile, but they also present alterations in angiogenesis. They have been shown to have delayed limb reperfusion after femoral artery ligation [104,105] and incomplete revascularization after myocardial infarction [91]. Moreover, the Matrigel^®^ plugs implanted in these mice are infiltrated by a lower amount of ECs than those implanted in wild type (WT) mice [104]. It has also been shown that the neovascularization of the retina after oxygen-induced ischemic retinopathy (OIR) presents defects, with few or no angiogenic tufts [93]. However, during the physiological development of the retina of these mice, a high number of proliferating ECs that lead to a more branched vasculature has been observed [106]. There is another endoglin-deficient animal model: Eng-iKO^e^ mice, which lack endoglin specifically in the endothelium. These mice have AVMs in the retinal vasculature in the first days after the birth. In addition, the vascular front is delayed and the veins are thickened [107,108,109].

When the effect of endoglin deficiency on the different phases of angiogenesis has been studied, it has been seen that aortic rings from *Eng*^+/−^ mice develop fewer sprouts than those from WT mice [93]. Furthermore, in a mosaic mouse containing endoglin-deficient and WT cells it was shown that, in the retinal vasculature, the amount of endoglin-deficient tip cells was much lower than that of WT cells [90]. However, if the mosaic was formed by WT and endoglin-overexpressing cells, these were preferentially located in the sprouting region [90]. It has also been shown that low endoglin levels inhibit the formation of pseudocapillars in vitro [88,104,110].

The main cellular processes involved in these models are the proliferation and migration of ECs. There is some debate about the role of endoglin in proliferation. Some authors defend that it is an anti-proliferative protein, since its absence increases the endothelial proliferation in vitro [111,112,113]. Other studies show that *Eng*^+/−^ ECs grow more slowly than WT cells, suggesting a pro-proliferative effect of endoglin [104,114]. Moreover, the treatment with antisense endoglin oligonucleotides increases the anti-proliferative response to TGF-β [88]. On the other hand, endoglin is located at focal adhesion and regulates cell migration [115] due, at least in part, to its ability to bind zyxin [116] and the change of location of ZRP-1 [117], thus participating in the cytoskeleton reorganization. Therefore, changes in endoglin expression can alter cell migration. However, the role of endoglin in this process is not clear. First, blood outgrowth endothelial cells (BOECs) and endothelial progenitor cells (EPCs) from HHT-1 patients present a reduced mobility with respect to cells from healthy donors [118,119,120]. In addition, the migration of human umbilical vein endothelial cells (HUVECs) treated with anti-endoglin antibody TRC105 is decreased [121]. In contrast, endoglin-deficient murine embryonic endothelial cells (MEECs) migrate more than control cells, but, when endoglin levels are restored, migration is reduced to the same levels as control cells [122,123].

It has also been shown that endoglin participates in the modulation of the endothelial barrier. Endoglin haploinsufficiency has been associated with increased weakness of the endothelial barrier, leading to increased vessel permeability [110,124]. In some cases, this permeability has been related to increased VEGF expression [93,125]. However, another study has shown that the augmented permeability observed in *Eng*^+/−^ and *Eng*^−/−^ cells is due to a decrease in VE-cadherin expression and the constitutive activation of RhoA [126].

Mural cell recruitment is also affected by reduced endoglin levels. In fact, a poor association of vascular smooth muscle cells to the endothelium is one of the causes of death in *Eng*^+/−^ mice during the embryonic development [96,98,127]. Although the retinas of *Eng*^+/−^ mice do not show differences in the number of mural cells with the control mice [106], maybe they cannot adhere correctly to the endothelium. Supporting this, Rossi and collaborators demonstrated that endoglin interacts with the integrins of the mural cells, thus favoring their adhesion to the endothelium and, therefore, the vascular maturation [110]. This suggests that the presence of immature vessels may also be a cause of recurrent epistaxis and internal bleeding in HHT patients [128].

All these studies, in which the effect of reduced levels of endoglin is mainly studied, lead to the conclusion that endoglin is a pro-angiogenic molecule, so the increase of its expression has been proposed as a treatment in diseases where angiogenesis is diminished [35]. However, a recent study has shown that continuous and ubiquitous overexpression of endoglin does not increase reperfusion after ischemia by femoral artery ligation and even inhibits the progression of the angiogenic front in the retinal vasculature. In addition, overexpression of endoglin maintains ECs in an active phenotype and prevents recruitment of mural cells to new vessels [34] (Figure 4A). For all these reasons, it does not seem that the uncontrolled increase of endoglin is a good therapeutic strategy.

This overexpression has been observed in the ECs of different solid tumors [95]. What is more, endoglin immunohistochemistry is used to measure microvascular density [129,130], and the high number of endoglin-positive vessels is associated with a worse prognosis [131,132,133,134,135] (Figure 4A).

As in physiologic angiogenesis, most studies examining the role of endoglin in tumor angiogenesis use endoglin-deficient mice. Tumors developed in *Eng*^+/−^ and Eng-iKO^e^ mice are smaller and less vascularized than those of control mice [136,137]. In addition, *Eng*^+/−^ mice develop fewer tumors when subjected to a skin carcinogenesis model, but the frequency of conversion of papillomas to carcinomas and the incidence of squamous cell carcinoma is higher in these mice, showing accelerated malignant progression [138]. Anderberg and collaborators demonstrated that haploinsufficiency of endoglin increases the frequency of metastases, probably due to an increase in vascular permeability [124]. In contrast, in a prostate cancer model, *Eng*^+/−^ mice have a higher tumorigenesis, but the tumors are smaller, less vascularized and generate less metastases [139]. There are also studies that analyze the incidence and survival of cancer in HHT patients. On the one hand, it seems that suffering from HHT does not affect the appearance of breast, lung, colorectal and prostate tumors [140]. On the other hand, a study involving HHT patients, relatives, and controls showed that endoglin deficiency reduces the incidence of solid tumors, especially breast cancer [141]. Regardless of whether or not the deficiency affects incidence, it appears that once the tumor is present, HHT patients have a longer survival [142].

It has also been shown that CD105^+^ renal tumor cells release microvesicles or exosomes loaded with proangiogenic mRNA and miRNA that induce angiogenesis in vitro and in vivo and the creation of a premetastatic niche in the lung [66]. Contrary to expectations, mice that overexpress endoglin do not develop bigger or more vascularized tumors in a xenograft model. However, the lack of stability and maturation of the vessels produced by the endoglin overexpression increases the presence of intratumor hemorrhages and the number of circulating tumor cells and pulmonary metastases [34]. This incorrect perfusion could lead, as discussed above, to an immunosuppressive microenvironment, which, along with the increased frequency of metastases, could be responsible, at least in part, for the worse prognosis associated with tumors with high endoglin levels (Figure 4A).

### 4.2. Endoglin in Inflammation

Endoglin also plays an important role in other processes, such as the regulation of the immune response (Figure 4B). It has been seen that HHT patients have a lower number of lymphocytes than healthy controls [143]. They also have a higher risk of infections, probably due to defects in monocyte oxidative burst and phagocytosis [144] or an impaired homing of the immune cells to the damage tissues [145].

Some of these alterations may be due to the expression of endoglin in different types of immune cells [67,71]. In fact, in 1985 endoglin was identified as a protein expressed in pre-B leukemia cell line [146]. Currently, it is known that it is constitutively present in most of the memory T lymphocytes and in 30% of the naïve T cells [71]. Recently it has been shown that it is also expressed in intratumor Treg lymphocytes of patients and mice [72], which could support the involvement of endoglin in the creation of an immunosuppressive TME. However, endoglin, which is also expressed in monocytes and in different phases of their differentiation [74,75], seems to stimulate the polarization of TAMs towards a M1 phenotype [69,80,147,148,149]. Aristorena and collaborators even demonstrated that L-endoglin promotes the M1 phenotype, while S-endoglin potentiates the M2 [80]. It has also been shown that the haploinsufficiency of endoglin in monocytes reduces their migration capacity [150] and that the lack of endoglin in macrophages impairs their phagocytic activity, leading to infections in vivo [69].

Endoglin also regulates the expression and activity of cyclooxygenase-2 (COX-2), since there is an increased expression of this protein in *Eng*^+/−^ mice [151]. COX-2 is usually overexpressed in tumors because it is released by CAFs, M2 macrophages, and other cells of the TME. It has an immunosuppressive role, since it is responsible for the generation of prostaglandin E2 (PGE2) that stimulates angiogenesis and tumor progression by inhibiting the activity of cytotoxic T lymphocytes [6,152].

Other alterations in inflammation may be due not to a deficit of endoglin expression in the immune cells themselves, but to a lack of endoglin in ECs. It has been shown that endothelial endoglin can bind to leukocyte integrins and regulate their extravasation by transendothelial migration, and that this process can be inhibited by the presence of soluble endoglin [153]. Regulation of extravasation by endoglin may also account for lower IL-6 and IL-10 expression and M2 macrophage infiltration seven days after tumor implantation in tumors from Eng-iKO^e^ mice [137]. However, these authors observed that interleukin expression and TAM infiltration increases at 14 days, and they proposed that this increase of TAMs may be the cause of the failure of anti-endoglin therapies [137].

Despite the controversies and contradictions, these data demonstrate that endoglin expression in immune and endothelial cells plays an essential role in the regulation of the inflammation.

### 4.3. Endoglin and Cancer-Associated Fibroblasts (CAFs)

It has also been shown that endoglin has a key role in CAFs (Figure 4C). In prostate cancer, CD105^+^ CAFs actively participate in the disease progression and resistance to androgen signaling deprivation therapy (ADT) by SFRP1 expression [62]. In animal models of this tumor type it has been shown that endoglin haploinsufficiency produces less accumulation of CAFs [139]. Endoglin also plays an important role in colorectal cancer. The expression of endoglin in CAFs of patients with stage II colorectal cancer correlates with increased development of metastases [61]. It also increases in vitro invasion and metastases and tumor invasion in colorectal cancer models in zebrafish and mice [61].

In addition to being expressed in CAFs, endoglin is one of the characteristic markers of MSCs, also present in tumors. In some cases, such as gastric cancer, CD105^+^ spindle-shaped stromal cells are associated with a worse prognosis [154]. CD105^+^ stromal cells with large migratory capabilities were also identified in breast cancer. These cells could be useful in predicting recurrence and metastasis [155].

## 5. Anti-Endoglin Therapies

Everything that is known about endoglin, especially in relation to tumors, has been used to develop anti-endoglin therapies. The most successful one is TRC105, a chimeric IgG1 monoclonal antibody developed by TRACON Pharmaceuticals that binds to the extracellular domain of endoglin and prevents BMP-9 binding. It has been shown to maintain ECs in a quiescent state and inhibit angiogenesis in vitro [35,156]. It also reduces the expression of VEGF and PDGF [121] and increases the release of soluble endoglin, which may represent an additional antiangiogenic mechanism [132]. Studies with this antibody have shown that it not only affects angiogenesis, but also reduces circulating Treg cells [157] and even targets CAFs [61,62] and other CD105^+^ cells of TME [158]. It also reduces circulating tumor cells and the generation of metastases [61,157,159]. In the clinic, the results obtained with the administration of TRC105 are promising in different types of cancer and especially if combined with other antiangiogenic drugs [132,160,161,162,163]. It has even reached phase III trials in angiosarcoma, although so far no clinical benefits have been observed [73]. Moreover, some studies suggest that conjugation of TRC105 with some drugs such as the ribosomal toxin nigrin B may be useful [132].

Bispecific liposomes have also been designed against FAP, expressed by CAFs, and endoglin, which contain high concentrations of a self-quenching near-infrared fluorescent dye, DY-676-COOH. In the clinic they can be useful to detect invasive margins or suspicious lymph nodes. However, if the encapsulated dye is replaced by a drug, it can be a powerful therapeutic strategy [164]. In fact, it has already been demonstrated that the encapsulated doxorubicin in these liposomes is more effective in vitro than when it is inside liposomes directed against a single target [165].

Gene therapy against endoglin using siRNA or shRNA is also promising. Anti-endoglin siRNA lipotransfection in ECs reduces proliferation and pseudocapillary formation in vitro. When siRNA is electrotransfected into subcutaneous tumors in mice, growth is retarded and the number of blood vessels is reduced [166]. However, it has the disadvantage of having a very short half-life. Expression of an anti-endoglin shRNA under a constitutive (U6) or endothelial (endothelin-1) promoter reduces proliferation, migration, invasion, and the ability to form pseudocapillaries in vitro [167,168]. In addition, if shRNA is introduced into the tumor by gene electrotransfer (GET), necrotic areas are increased and the number of vessels is reduced [167]. If the tumor cells express endoglin, shRNA treatment also reduces proliferation and spheroid growth in vitro and tumor growth in vivo. In addition, a high number of mice were tumor free 100 days after treatment [168,169]. Unfortunately, although treatment with siRNA or shRNA does not produce systemic toxicity, several consecutive administrations are necessary for the treatment to be effective [166,170], so they may be more useful as an adjuvant to chemotherapy and radiotherapy [170].

Finally, the results obtained with the use of endoglin-based DNA vaccine should be highlighted. This therapy activates the specific and non-specific immune response against vessels and tumor cells, inhibits angiogenesis, reduces the M2 phenotype of TAMs, induces the recruitment of CD8^+^ and CD4^+^ lymphocytes, and inhibits primary tumor growth and metastases. In addition, it has the advantage over other treatments that it is administered orally. All these results are enhanced if the endoglin-based DNA vaccine is administered together with IL-12 [8,171].

## 6. Conclusions and Future Perspectives

From all the information presented in this review, it can be concluded that TME is a pivotal influence for tumor prognosis, since immunosuppressive TME present worst prognosis and respond poorly to therapies. In this context, endoglin appears as a crucial molecule in the determination of an immunosuppressive TME, mainly because of its role on angiogenesis but also in inflammation and in CAFs biology (Figure 4).

Therefore, the analysis of endoglin expression in patient biopsies could be an excellent biomarker of immunosuppressive TME rather than increased angiogenesis. This may predict the patient’s response to immunotherapy, allowing to decide if its administration is worthy. In fact, although it has not been experimentally analyzed, an algorithm has shown that endoglin is one of the possible biomarkers of immunosuppressive TME in hepatocellular carcinoma. The aim is to use a panel that includes endoglin and other biomarkers to determine clinical outcome and administer more personalized treatment [172]. It would be very interesting and highly recommended to test the usefulness of this model in different tumor types, including desmoid and hypervascular tumors, which have a large number of CAFs and ECs, respectively.

However, an even more exciting perspective is the use of endoglin as a therapeutic target allowing vascular normalization and, consequently, the generation of a more immunosupportive TME. We hypothesize that, similar to what has been observed with anti-VEGF therapies, the administration of adequate concentrations of anti-endoglin treatments could decrease endoglin to levels that would allow the normalization of the vessels, but in which the adverse effects that have been observed in some endoglin-deficient animal models would not be produced. This would be supported by the fact that endoglin over-expression inhibits the stabilization and maturation of the vessels [34]. Thus, the vascular normalization generated by anti-endoglin treatment would improve blood flow and tumor perfusion, which would reduce hypoxia, promote CD8^+^ lymphocyte activation, and favor the transformation of M2 TAMs into M1 TAMs. All this would result in the transition from cold to hot tumors.

Moreover, as stated in previous sections, endoglin is expressed in other cells of the TME that can promote tumor growth, such as CAFs, Tregs, or MSCs. In this context, anti-endoglin therapy would have vasculature-independent effects that would contribute to the modification of the TME composition towards a more immunosupportive phenotype. In fact, studies with the TRC105 antibody have already demonstrated some of these effects, such as modifying the invasiveness and survival of CAFs [61,62] and reducing circulating Tregs [157], but there are other potential targets yet to be explored.

In summary, the role of endoglin in the regulation of the TME is really promising and its in-depth study will lead to breakthroughs in cancer treatment but also in predicting the response to therapies that are currently being used with limited results.

## Data Availability

No new data were created or analyzed in this study. Data sharing is not applicable to this article.

## Abbreviations

ADT	androgen signaling deprivation therapy
ATP	adenosine triphosphate
ATX	autotaxin
AVM	arteriovenous malformation
BOEC	blood outgrowth endothelial cell
CAF	cancer-associated fibroblast
COX-2	cyclooxygenase-2
DNA	deoxyribonucleic acid
EC	endothelial cell
ECM	extracellular matrix
EMT	epithelium-mesenchyme transition
EPC	endothelial progenitor cell
FAP	fibroblast activation protein
GET	gene electrotransfer
HHT	hereditary hemorrhagic telangiectasia
HUVEC	human umbilical vein endothelial cell
IFN	interferon
IL	interleukin
LPA	Lysophosphatide
MDSC	myeloid-derived suppressor cell
MEEC	murine embryonic endothelial cell
miRNA	micro ribonucleic acid
mRNA	messenger ribonucleic acid
MSC	mesenchymal stromal cell
NK	natural killer
OIR	oxygen-induced ischemic retinopathy
PDGF	platelet-derived growth factor
PDT	photodynamic therapy
PET	positron emission tomography
PGE2	prostaglandin E2
ROS	reactive oxygen species
shRNA	short hairpin ribonucleic acid
siRNA	small interfering ribonucleic acid
S1P	sphingosine-1-phosphate
TβRI	TGF-β receptor I kinase
TβRII	TGF-β receptor II kinase
TAM	tumor-associated macrophage
TGF-β	transforming growth factor β
TME	tumor microenvironment
VEGF	vascular endothelial growth factor
VEGFR	VEGF-receptor
WT	wild type
